# Adaptive Radiation Therapy for Head and Neck Cancer—Can an Old Goal Evolve into a New Standard?

**DOI:** 10.1155/2011/690595

**Published:** 2010-08-18

**Authors:** David L. Schwartz, Lei Dong

**Affiliations:** ^1^Department of Radiation Medicine, North Shore-LIJ Health System, 270-05 76th Avenue, New Hyde Park, NY 11040, USA; ^2^Department of Radiation Physics, University of Texas MD Anderson Cancer Center, Unit 94, 1515 Holcombe Blvd., Houston, TX 77030, USA

## Abstract

Current head and neck intensity-modulated radiotherapy (IMRT) techniques cause significant toxicity. This may be explained in part by the fact that IMRT cannot compensate for changes in the location of disease and normal anatomy during treatment, leading to exposure of at-risk bystander tissues to higher-than-anticipated doses. Adaptive radiotherapy (ART) is a novel approach to correct for daily tumor and normal tissue variations through online or offline modification of original IMRT target volumes and plans. ART has been discussed on a conceptual level for many years, but technical limitations have hampered its integration into routine care. In this paper, we review the key anatomic, dosimetric, and treatment delivery issues at play in current investigational development of head and neck ART. We also describe pilot findings from initial clinical deployment of head and neck ART, as well as emerging pathways of future research.

## 1. Introduction

Head and neck intensity-modulated radiotherapy (IMRT) holds promise to provide excellent locoregional control of head and neck cancer while sparing salivary function and dose to normal structures [1–3]. IMRT utilizes 3D anatomic information extracted from imaging acquired several days prior to treatment. It is recognized that the location, shape, and size of disease and normal anatomy change significantly due to daily positioning uncertainties and physiological factors during a 6-7-week course of treatment. These changes include regression of primary tumor and nodal disease, alterations in normal glands and mucosa, resolution of postoperative soft tissue effects, and alterations in body habitus due to weight loss [4–8]. Such changes can be dramatic. Our group has used serial CT imaging during H and N treatment to demonstrate that primary tumors can shrink volumetrically by up to 90% and that parotid glands can involute and shift medially (towards high-dose coverage in the oropharynx) by up to a centimeter during a treatment course [8]. 

Due to clear dosimetric advantages enjoyed by IMRT over conventional techniques, the radiation oncology community has adopted routine use of IMRT for head and neck cancer in the absence of prospective confirmation of clinical impact. Only recently has randomized data from Europe been presented in abstract format (C. Nutting et al., *Proc. ASCO* 2009) to demonstrate that IMRT reduces parotid toxicity, albeit with little benefit beyond this relative to conventional therapy. 

Hands-on experience confirms that IMRT continues to cause severe acute oral and pharyngeal side effects [1–3]. Much of IMRT's ability to reduce toxicity remains potentially unrealized when statically guided by pretreatment imaging. Treatment failures may also result from unanticipated tumor movement across critical regions of sharp dose gradient. There are potential advantages to manual replanning of IMRT midway through treatment [9], but to date no prospective clinical study has been performed to demonstrate the clinical benefits and practicality of this approach. In addition, the ideal timing for replanning remains an unresolved issue.

Adaptive radiotherapy (ART) is an approach to correct for daily tumor and normal tissue variations through online or offline modification of original IMRT target volumes and plans. It is a treatment strategy tightly linked to feedback-based control theory. ART has three basic components: (1) detection of changes, (2) method of intervention, and (3) management of overall clinical goals. Successful implementation of each component determines the overall success of clinical application. ART is a decades' old concept, but technical limitations have held back integration of ART into routine care. Older ART approaches have depended largely upon implanted fiducial markers or infrequent serial imaging to guide laborious manual replanning. Ideally, direct staff input must be replaced by automated processes to make ART practical. Exploratory work [10–12] has demonstrated the feasibility of ART planning for prostate and H and N cancer using daily in-room CT image guidance to localize targets. A prospective clinical trial testing the feasibility of an automated H and N ART delivery approach at M.D. Anderson Cancer Center is currently underway; initial results from this work will be described further below.

## 2. Clinical Rationales and Typical Treatment Approaches for H and N Radiotherapy

One key requirement for IMRT planning is target definition [13–17]. Gross target volume (GTV) determines the anatomic region which harbors the highest tumor cell density and requires the highest prescribed dose. Target definition is also important to minimize dose to normal bystander anatomy. Postradiotherapy salivary production is well predicted by dose-volume effects [18, 19]. Dysphagia and aspiration are related to irradiated volumes for certain functional structures [20, 21]. IMRT can potentially allow clinicians to meet defined parotid and dysphagia-related dose constraints and improve objective and subjective measures of toxicity [1, 2, 22]. However, given that IMRT's steep dose gradients could potentially move across shifting boundaries between neighboring disease and avoidance structures, meaningful improvements in therapeutic ratio may only be attained with accurate knowledge of true target locations. As will be discussed below, lack of updated targeting during a course of therapy is a likely culprit responsible for continued issues with treatment toxicity during and following IMRT.

## 3. Margins and Treatment Designs—Balancing the Risk and Benefit

Planning target volumes (PTVs) are used to compensate for treatment setup uncertainties through volumetric expansion of CTV margins. PTV expansions present dosimetric tradeoffs since these frequently overlap geographically with adjacent at-risk normal structures. A favorable therapeutic index relies on accurate knowledge of setup uncertainties, normal organ dose tolerances, and delineation of treatment targets to safely minimize PTV expansions. Questions surrounding optimal head and neck radiotherapy treatment design and delivery include the following.

- What are the characteristics and kinetics of setup errors in the H and N region?
- How quickly and by how much can tumor and normal anatomy change during treatment?
- What are the dosimetric consequences of anatomic changes and setup errors?
- What is a practical workflow template for H and N ART to compensate for these uncertainties and to reduce need for PTV expansions?
- Is there evidence that ART can be feasibly deployed? What are the most pressing (or most surmountable) hurdles impeding future widespread deployment of clinically meaningful H and N ART?

## 4. H and N Setup Uncertainties

### 4.1. Baseline Anatomic Uncertainties

Setup uncertainties relevant to H and N radiation treatment have been actively investigated [23–30]. Several setup correction protocols have been proposed to improve corrections for such uncertainties [31–35] that occur despite the use of custom-fitted thermoplastic masks and head rests. With the recent availability of kV X-rays mounted orthogonally relative to the therapy beam line, it is possible to acquire high-quality images for daily image guidance [36]. An advantage of in-room kV imaging is that it provides images similar to digitally reconstructed radiographs (DRRs) derived from simulation CTs. However, this 2D method assumes that anatomic landmarks are imaged and measured identically across time, which may not be true. 

In-room CT scanners, tomotherapy-based megavoltage CT, and gantry-mounted cone beam CT are now all available to provide in-room 3D imaging [37–40]. A unique requirement for 3D position verification is selection of a region of interest (ROI) to determine shifts relative to reference simulation images. This process is complicated by semi-independent movement of the body constituents of the H and N region. The skull is attached to a semirigid mandible and to a column of cervical vertebral units with multiple degrees of movement freedom [41]. Zhang et al. [42] were the first to use in-room CT imaging and serial 3D image registration to analyze the relative movement of different H and N ROIs. Three bony ROIs were defined: C2 and C6 vertebral bodies and the palatine process of the maxilla (PPM) as a surrogate for the skull base. Although ROI movement shifts were highly correlated to a clinical isocenter marked on the immobilization mask, displacement uncertainties of up to 2–6 mm were observed between any two ROIs, indicating the effects of flexibility and rotation on position uncertainty. In a more recent study [43], eight ROIs were defined and analyzed in 38 patients who had serial cone-beam CT imaging taken during treatment. Different subregions were shown to move differently due to differential flexion across regions. Local residual errors ranged up to 3.4 mm (one standard deviation), translating to a need for 4–7 mm PTV margin expansions even despite daily image guidance.

The site of the largest systematic setup uncertainty in H and N region is the larynx, emphasizing the importance of internal motion secondary to swallowing and tongue movement. Unfortunately, such internal movement cannot be directly addressed in patients (at least not comfortably). Because the larynx and tongue are attached to the hyoid bone, tongue movement may result in displacement of the larynx. Inspiration and expiration also causes displacement of the larynx, although this displacement is usually modest at rest. Videofluoroscopy can demonstrate 20–25 mm cranial-caudal and 3–8 mm anterior-posterior laryngeal movement during swallowing of liquid [44–46]. Fortunately, the proportional duration of active swallowing is low and does not appear cause dosimetric deviations during treatment [47].

**Figure 1:**
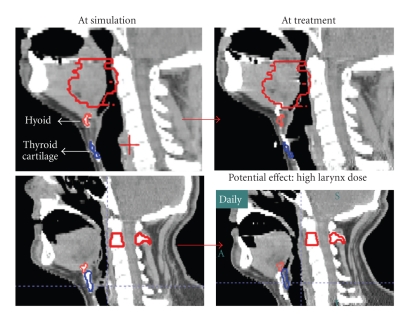
The positions of the hyoid and thyroid cartilages can change noticeably during the simulation or treatment delivery. Because the swallowing action is usually infrequent and has a short duration, a simulation CT could be biased towards an infrequent anatomical pose by the consequence of swallowing. If the hyoid and thyroid cartilages are captured at their most inferior positions (top row), the larynx may receive higher dose during treatment. In the converse situation, if the hyoid and thyroid cartilages are captured at their most superior positions during CT simulation (bottom row), a primary target near the base of tongue could be underdosed during treatment.

Work at M.D. Anderson Cancer Center has investigated internal systematic and random errors caused by swallowing using a fast helical CT scanner (CT-on-Rails), measuring the position of the thyroid cartilage relative to that of the hyoid bone in 17 oropharyngeal cancer patients. A total of 555 daily CTs were taken (30–33 images/patient). These structures were found to move up to 1.6 cm during a treatment course relative to vertebral landmarks, most notably in the superior-inferior (SI) direction. Systematic errors relative to baseline position from simulation for individual patients were large (up to 12 mm). The clinical significance of this error is illustrated in Figure 1. Because swallowing is infrequent and has a short duration (~1 second), its intrafractional dosimetric effect is less critical than its systematic impact. For example, a fast simulation CT could be heavily biased by an infrequent anatomical pose if obtained mid-swallow. In such an instance, the larynx may receive higher dose during treatment if the hyoid bone and neighboring oropharyngeal sites targeted for high dose are inadvertently captured at their most inferior position (top row). In an opposite situation, if the hyoid and thyroid are captured at their most superior position during CT simulation (bottom row), a primary target near the base of tongue could be underdosed during treatment.

### 4.2. Anatomic Uncertainties Occurring during Treatment

**Figure 2:**
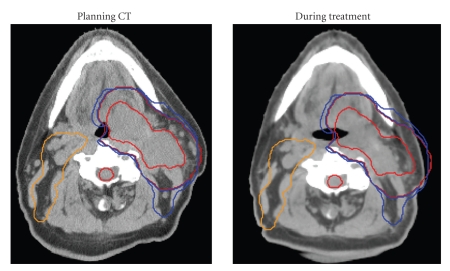
Anatomic changes can be pronounced during treatment. In this example, planning CT scan and CTV contours are shown on the left. On the right, a mid-course CT (three weeks into treatment) demonstrates significant reduction in gross tumor (thick red line). Baseline CTVs have been overlaid via rigid image registration. These match current anatomy poorly and in fact extend past the skin contour into air.

Some patients receiving radiation therapy to the head and neck region will have significant anatomic changes during their course of treatment, including shrinking primary tumors or nodal masses, resolving postsurgical edema, and weight loss [4–7]. An example of such a case is shown in Figure 2. This illustrates how common on-treatment anatomical changes render an original IMRT plan to be less conformal than its original intent.

**Figure 3:**
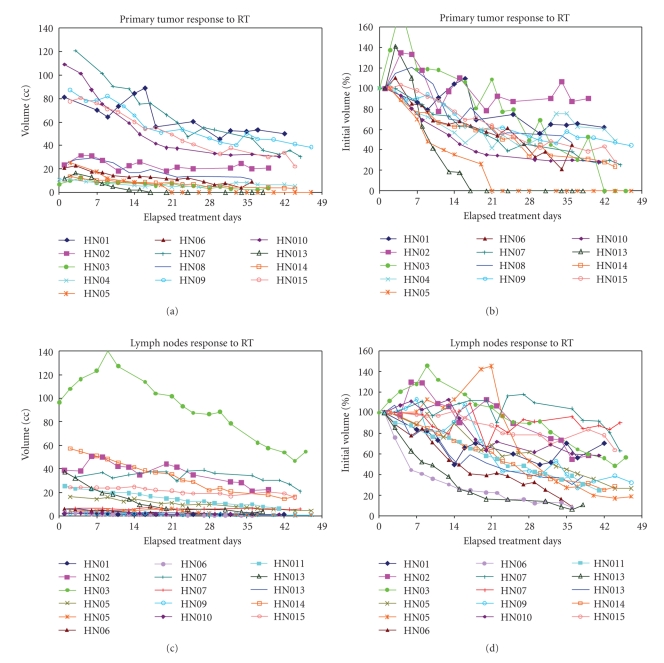
Gross tumor volume changes over time among patients with head and neck cancers. Both primary tumor (a) and (b) and lymph nodes greater than 2 cc of volume (c) and (d) are showing similar trend. The gross tumor volumes decreased at a median rate of 0.2 cc or 1.8% of initial volume per treatment day. (Reprinted from [8]).

Barker et al. [8] used serial in-room CT imaging to study gross tumor volume (GTV) changes during a complete treatment course. CT scans were acquired three times weekly in 14 patients. Manual contouring was used to evaluate GTV changes. GTV decreased throughout therapy at a median rate of 0.2 cc per treatment day (range, 0.01–1.95 cc/day). Figure 3 shows the volume change for primary tumors and lymph nodes. It was found that both primary tumors and involved lymph nodes lost volume at approximately the same rate of 1.7%-1.8% per treatment day. On the last day of radiation treatment, this corresponded to a median total relative loss of approximately 70% of the initial GTV (range, 10%–92%). Rate of volume loss was strongly associated with the baseline target volume, a relationship which could help to identify candidate patients who may benefit most from an adaptive radiotherapy approach.

**Figure 4:**
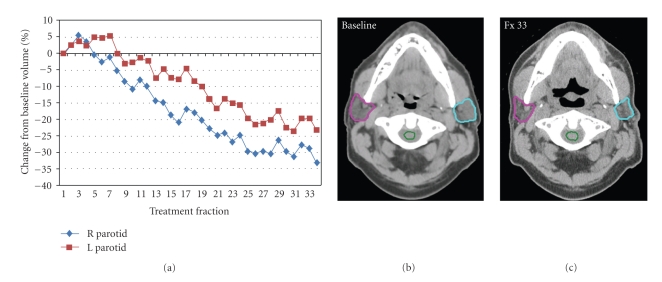
A case example of changes in parotid gland volume during a 33-fraction IMRT treatment course. (a) shows the percent of volume change for each parotid as a function of treatment fraction. The (b) and (c) shows an axial CT slice of the parotid before radiotherapy (b) and after 33 fractions of radiotherapy (c).

Parotid glands also involute during therapy (Figure 4). Barker et al. observed that the median parotid volume loss was 0.2 cc/day or 0.6%/day of the initial volume. At the end of treatment, median parotid volume loss was 28.1%. The center of mass of both parotid glands shifted medially over time. By the end of treatment, this medial shift was 3.1 mm (range: −0.3–9.9 mm). Lee et al. acquired similar data using daily megavoltage CT imaging [48]. Day-to-day variations in the center-of-mass distance and volume were 1.61 mm and 4.36%, respectively. Parotid volumes decreased with a median total loss of 21.3% and a median change rate of 0.7%/day. Parotids migrated toward the patient center with a median total distance change of −5.26 mm (0.00 to −16.35 mm) and a median change rate of −0.22 mm/day. Another CT-based study of 82 head and neck cancer patients showed an average volume loss in parotid glands of 20.0%, 26.9%, and 27.2% after 3-week mid-treatment, at treatment completion, and 2-month posttreatment, respectively [49]. These gland volume reductions correlated significantly with mean dose to the irradiated glands: volume loss with higher mean parotid doses (>30 Gy) to the glands was significantly greater than for lower mean parotid doses (*P* < .001). 

Finally, nearly all patients lost weight throughout their course of treatment. Barker et al. found that the median weight change from the start to completion of treatment was –7.1% (range, +5.2% to –13.0%) in their study. Reductions in external skin contours at the level of the C2 vertebral body and at the base of skull correlated with weight loss. Median weight loss correlated significantly with median parotid medial displacement over time (*P* < .001). This confirms that skin contours and weight loss can potentially be used as easy-to-measure harbingers of underlying anatomy changes.

## 5. Image-Guided Adaptive Radiotherapy for H and N Cancer

### 5.1. Image-Guided Approaches for H and N Radiotherapy

The term image-guided radiation therapy (IGRT) is commonly interpreted as the use of in-room imaging to make setup corrections, in particular, positional shifts. Image guidance does not typically involve modification of the original treatment plan, which means that IGRT aims at correction of setup errors and reducing CTV-to-PTV margin. Although there are other approaches for H and N patient setup, in-room stereoscopic and volumetric imaging are the most commonly used techniques for IGRT.

**Figure 5:**
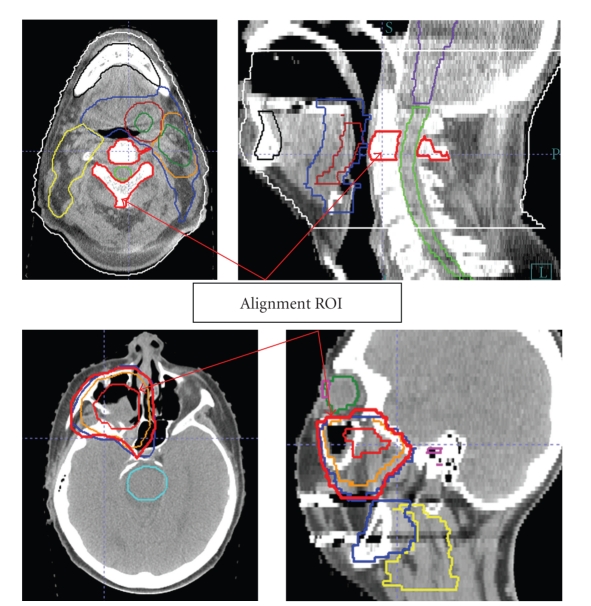
Region-of-interest (ROI) selection for H and N IGRT should be based on clinical goals and is ideally located in proximity to critical clinical target volumes and/or normal structures requiring strict sparing. The top row shows a base-of-tongue cancer in which the C2 vertebral body was selected as an alignment object close to both GTV and spinal cord. For a sinus cancer case (bottom row), the PTV was used as the alignment ROI for patient setup to optimize coverage of high-risk CTVs and sparing of closely neighboring neural tissues.

IGRT workflow for H and N patients is not different from other treatment sites, with the notable exception that an ROI should be explicitly identified by the treating physician as a part of patient's treatment directive. Previous studies have provided sufficient evidence that the relative movement of different ROIs can be significant in the H and N region. Ignoring these differences in different regions would introduce additional setup uncertainties [36, 43]. The selection of ROI depends on the treatment case, which is a balance between target coverage and normal structure sparing. Typically, the spinal cord sparing is critical for most H and N cases; therefore, a vertebral body is generally a good choice as a reference ROI for alignment. For an oropharyngeal cancer case, a high cervical (C1–C3) vertebral body is a reasonable choice (Figure 5). Although a PTV can be used in particular cases, one should be cautious using GTV as an alignment target since asymmetric tumor shrinkage can affect the geographic relationship of GTV to isocenter and avoidance structures.

### 5.2. Anticipated Workflow for Image-Guided Adaptive Radiotherapy

In contrast to image-guided setup for repositioning treatment fields, the intent of ART is to appropriately modify a radiation treatment plan to account for temporal changes in anatomy. In theory, ART can occur at three different timescales: offline between fractions, online immediately prior to a fraction, and in real-time during a treatment fraction. ART can be tightly linked to image guidance processes because any volumetric images acquired for IGRT procedure could also be used for monitoring changes in anatomy and designing new plans.

**Figure 6:**
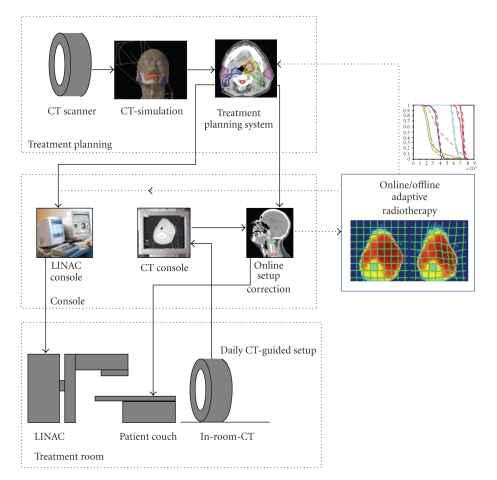
A workflow diagram for in-room CT or CBCT-guided adaptive radiotherapy. The first level of treatment modification is a simple couch shift to correct for daily setup errors (CT-guided IGRT). Nonrigid changes in tumor volumes and normal organs can then be corrected via an online or offline adaptive replanning process (dotted lines).

An example of ART workflow is shown in Figure 6. Daily in-room volumetric imaging is essential to both the IGRT and ART pieces of this workflow. Solid lines indicate the image-guidance procedure controlling the position of the treatment couch. Volumetric images can be simultaneously sent to a treatment planning system where a new treatment plan can be adapted to current anatomy via automated deformable registration software and sent back to the therapy machine for delivery. This is shown in dotted lines in the procedure workflow. The adapted plan could be either deployed immediately (online correction) or used for future treatment (offline correction).

### 5.3. Deformable Image Registration for Autosegmentation

Manual segmentation of treatment planning images demands too much physician and staff effort to be practical for routine deployment of ART. Manual contouring would also be susceptible to intra- or interobserver variations [50], which could adversely affect the consistency of treatment quality. Deformable image registration for atlas-based autosegmentation is an effective alternative for serial adaptive replanning [14, 51–55]. Deformable image registration is a geometric mapping process that creates one-to-one correspondence between two images of the same object deformed across time. If the contours exist in one of the reference CT images, deformable transformation can be used to transform reference contours onto the newly acquired CT images with minimal manual input. This is well suited for ART, given that the original treatment plan can serve as the reference for this process.

**Figure 7:**
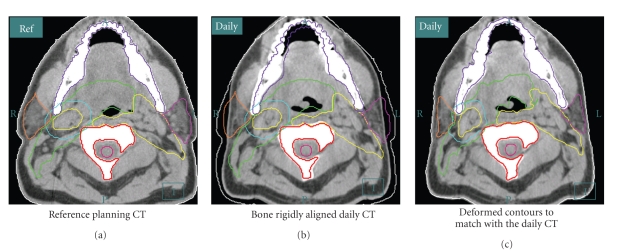
The ART process for patient treatment starts with a rigid alignment (in this example, to the C2 vertebra) between the reference planning CT and the daily in-room CT ((a) and (b)). The planning contours are overlaid to the daily CT to verify setup accuracy and to evaluate if there are changes in current anatomy relative to baseline. If the changes are significant, as illustrated in (b), a deformable image registration can be performed to propagate original planning contours onto current anatomy. The resultant contours are shown on (c). This process takes less than 30 seconds.

An example of using deformable image registration for autosegmentation is shown in Figure 7. The process starts with a rigid alignment of bony structure (C2 vertebra) between the reference planning CT (left) and the daily in-room CT (middle and right). The necessary planning contours are overlaid onto the daily CT to verify setup accuracy and to ascertain whether significant anatomic changes have occurred at interval. If the changes are significant (for example, the original clinical target volumes no longer adequately cover gross disease visualized on daily CT images), deformable image registration can be performed to propagate the planning contours to the daily anatomy. The resultant contours are shown to the right. The entire transformation takes seconds, making it relevant to either online or offline IMRT replanning. Manual physician recontouring can take several hours [14], which would not be practical for online ART and would strain practical application of offline ART.

### 5.4. Dosimetric Benefits of H and N ART

With deformable image registration for dose accumulation, it is possible to evaluate uncorrected IMRT relative to IGRT or full ART. O'Daniel et al. [56] studied the differences between planned and delivered parotid gland and target doses in a group of H and N cancer patients receiving standard IMRT. The clinical IMRT plans, designed with 3 mm to 4 mm PTV margin expansions, were recalculated on the repeated CT images. Deformable image registration software was used to map daily dose distributions to the original treatment plan and to calculate a cumulative delivered dose distribution. Without IGRT, dose to the parotid gland increased above the planned dose by 5–7 Gy in 45% of the patients. Use of IGRT aligned to the C2 vertebral body provided modest but significant parotid dose reductions in 91% of patients (median, 2 Gy). Nonetheless, the parotid dose from bone alignment remained greater than planned doses (median, 1.0 Gy, *P* = .007) due to parotid shrinkage and movement.

Using daily MV imaging in 10 tomotherapy patients, Lee et al. analyzed changes in parotid gland dose using a deformable image registration method [57]. They found that the daily parotid mean dose of the 10 patients differed from the plan dose by an average of 15%. At the end of treatment, 3 of the 10 patients were estimated to have received a greater than 10% higher mean parotid dose than in the original plan (range, 13%–42%). Dose differences correlated with medial drifting of the parotids toward the high-dose region. 

Wu et al. performed a comprehensive adaptive replanning simulation study to evaluate the differences between planned and delivered doses and to investigate different replanning strategies [58]. Eleven patients underwent six weekly helical CTs during routine IMRT. Cumulative doses to CTVs were preserved even with use of 0 mm PTV expansion margins. Significant increases in parotid dose were observed without adaptive replanning. The authors reported that one adaptive replanning during midcourse improved parotid mean dose sparing by 3%, two replannings by 5%, and six replannings by 6%, assuming that adaptive replanning transpires one week prior to actual treatment delivery. If six weekly replans are used immediately, parotid dose sparing is improved 8%. However, these calculations assumed that each new plan is executed without additional setup errors or nonrigid changes.

### 5.5. Clinical Experience with Automated H and N ART

Is automated H and N ART feasible, and what is its clinical impact? At M.D. Anderson Cancer Center a prospective, IRB approved clinical trial is underway to test these questions in oropharyngeal cancer patients treated with definitive IMRT. Patients with locally advanced AJCC stage III-IV disease are eligible for inclusion. Baseline IMRT planning follows standard guidelines, with volumetric CTV-to-PTV expansions of 3-4 mm. In-room CT-guided IGRT is used for each treatment session. This rigid alignment step corrects for any setup errors based on the bony landmark of cervical vertebrae in the C1–C3 region. Attending physicians have the chance to evaluate each daily IGRT setup. If significant anatomic changes resulting in geographical miss of gross tumor or inadequate sparing of normal tissues (particularly parotid glands or larynx) are noted by a physician triaging the daily CT images, then formal dosimetry and planning evaluations are instigated. Deformable image registration is performed with a validated in-house version of Thirion's Demons algorithm [52] which is used to transfer the initial contours (GTV, CTV, parotid, spinal cord, brainstem, and other normal structures) to the new CT image set. In addition to contours, isocenter information and the original IMRT plan are loaded into a new plan based on the newly acquired CT image set. A copy of this baseline plan is made, and a new updated plan can then be designed based on current anatomy. Additional planning constraints may be added to improve the quality of the new plan on an individualized basis.

It is important to emphasize that this trial does not employ PTV expansions for adaptive replanning. Our pilot experience has confirmed highly precise treatment setup reproducibility with our CT-guided IGRT procedure once patients have acclimated to treatment. Additional experience has confirmed that standard 3-4 mm PTV expansion margins are too generous to maintain parotid dose sparing if daily image guidance is employed [56]. Other than an absence of PTV expansions, ART planning does not differ from standard IMRT planning practice. Therefore, the intent for ART is to recapitulate the treatment planning goals of the original IMRT plan as faithfully as possible, rather than to create novel planning guidelines. 

**Figure 8:**
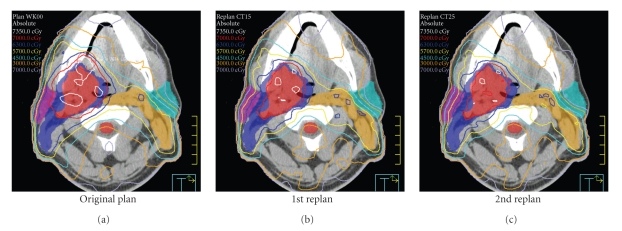
An example of serial ART dose recalculation using a daily CT image acquired at the 25th treatment fraction. On (a), the original plan is calculated on current anatomy. The original plan provides inappropriate treatment margins and dose heterogeneity within the high-dose CTV. In the (b), an earlier ART replan (ART1, designed at the 15th treatment fraction) is calculated onto current anatomy. On (c), a 2nd ART replan (ART2) is designed and calculated for the current daily image set. The ART2 plan provides improved contralateral parotid sparing and a lower total body dose than the ART1 plan.

An example of ART dose recalculation and replanning is shown in Figure 8. Figure 8(a), the original plan is calculated onto current anatomy. Due to loss of weight and tissue separation, there is less attenuation of each IMRT beam. As a result, the original plan provides inappropriately large treatment margins and considerable dose heterogeneity within the high-dose CTV. Figure 8(b), a previous ART replan (ART1, designed at the 15th treatment fraction) is calculated onto current anatomy. The ART1 replan significantly improves dose conformality because PTV expansions are not used. Figure 8(c), a 2nd ART replan (ART2) is designed and calculated for the current daily image set. The ART2 plan provides further improvement of contralateral parotid sparing and a lower scattered body dose relative to the ART1 plan.

**Figure 9:**
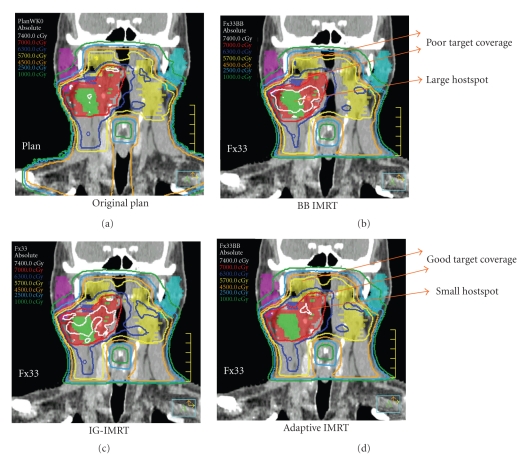
An example of cumulative dose evaluation for IMRT treatments without daily image guidance (alignment to the surface markers or “BBs”on the immobilization device) (BB-IMRT), image-guided IMRT (IG-IMRT), and image-guided adaptive IMRT described in this investigation (adaptive IMRT). Due to setup error, BB-IMRT has a tendency to underdose CTV.

**Figure 10:**
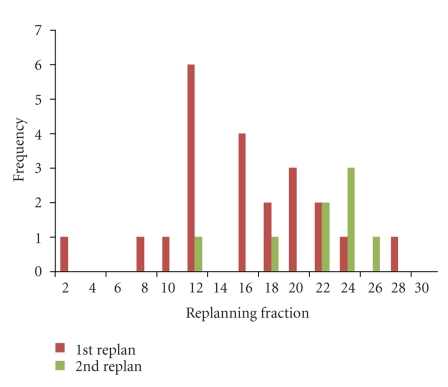
A histogram plot of the timing of the triggering fraction for replanning for the 1st replan and 2nd replan.

**Figure 11:**
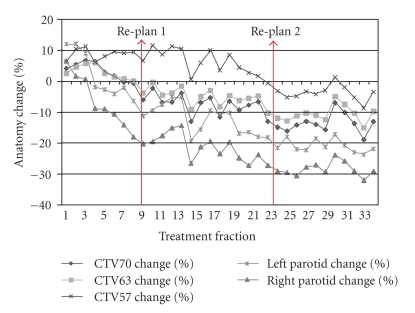
Volumetric changes for high-risk CTV (CTV70), intermediate-risk CTV (CTV63), low-risk CTV (CTV57), and parotid gland in a patient who had two replans performed during the course of treatment. The first replan occurred at the 9th treatment fraction, and the second replan was designed at the 23rd treatment fraction.

A preliminary analysis of 724 daily CT images in 22 patients who have completed a full course of ART for oropharyngeal cancer has been performed. The cohort consisted of 20 males and 2 females, with a median age of 58 years (range: 51–77). Primary disease site was base of tongue in 15 patients and tonsil in 7 patients. Nineteen patients had AJCC stage IV disease while 3 were of stage III. T stage distribution was 3 T1, 10 T2, 5 T3, and 4 T4 and N stage distribution was 3 N0, 2 N1, 3 N2a, 12 N2b, and 2 N2c. By treatment completion, mean parotid shrinkage was 26%, consistent with our original published findings [8]. All patients received at least one replan and 8 patients (36%) received two replans during their course of treatment. As illustrated in Figure 9, we compared 4 planning scenarios: (1) the original IMRT plan aligned to the marked isocenter (BB), (2) the original plan aligned according to daily bone alignment (IGRT), (3) IGRT with one adaptive replan (ART1), and (4) actual treatment received by each study patient (IGRT with either one or two adaptive replans, ART2). As summarized in Figure 10, the median trigger point for the first adaptive replanning was the 16th treatment fraction (range: 2 to 28), at which point combined parotid volumes had shrunk by 15% and combined CTVs had shrunk by 4% (see Figure 11 for a case example). For ART2 patients, the median trigger points for the first and second replanning were the 11th and the 22nd fractions, respectively. In retrospective dose analysis, ART1 group (16 cases) reduced the mean dose to contralateral parotid by 0.6 Gy or 2.8% (*P* = .003) and ipsilateral parotid by 1.3 Gy (3.9%) (*P* = .002) over the IGRT-only group. ART2 (4 cases) further reduced the mean contralateral parotid dose by 0.8 Gy or 3.8% (*P* = .026) and ipsilateral parotid by 4.1 Gy or 9% (*P* = .001). ART also significantly reduced integral body dose at 60 Gy and 40 Gy levels compared to IGRT alone.

This trial is the first prospective demonstration of the feasibility of adaptive head and neck radiotherapy. Significantly, initial results suggest that conventional IGRT alone does not provide meaningful dosimetric benefit if conventional PTV margins are used. One properly timed adaptive replan (ART1) appears to provide the most relevant dosimetric improvements. The study is ongoing and continues to correlate clinical outcomes to these dosimetric results.

## 6. Future Directions for ART Refinement and Deployment

### 6.1. Online Versus Offline Adaptive Radiotherapy

Online ART adjusts the treatment plan daily in real time using images acquired during each treatment session. An offline approach, on the other hand, uses imaging information from a previous time point for planning changes incorporated into a subsequent treatment session. The online approach can potentially provide greater treatment precision, at the cost of increased daily effort and treatment time. It is more challenging due to uncompromising time constraints (the patient remains immobilized on the treatment couch while waiting for ART corrections). In the case of H and N radiation treatment, most anatomic changes take place gradually over the first few weeks of therapy. There is no need for real-time intervention unless an acute, unforeseen event occurs, such as rapid disease progression. Therefore, in our opinion, offline adaptive radiotherapy appears to be a more practical approach for H and N cancer in the majority of cases, which should make ART easier to deploy across the practicing radiation oncology community.

### 6.2. Correction for Nonrigid Setup Error

In H and N radiotherapy patients, it is common for spinal anatomy to exhibit complex, nonrigid geometric changes that can affect treatment plan conformality. If these nonrigid changes are systematic (i.e., caused by impropriate simulation procedures), adaptive replanning can be used to correct these nonrigid systematic setup errors even if tumor target volumes have not changed. Our experience showed that random nonrigid setup errors are difficult to correct. Resetting patient's setup position usually does not fully correct these errors. In special cases (i.e., retreatment of areas close to critical structures, such as central nervous tissues) where desired accuracy requires daily real-time correction of random (nonrigid) setup error, online ART may become desirable.

### 6.3. Auto-Replanning

For all current ART strategies, replanning remains a time-consuming process. It will be necessary to reduce resource requirements for ART through effective automated planning techniques. Although replanning benefits from information provided by initial planning, trial-and-error is still necessary to fine-tune planning parameters for optimization. Several groups have studied auto-replanning algorithms. In almost all cases, deformable image registration is a critical component of this process. Mohan et al. [10] used an IMRT intensity warping technique to adapt an IMRT plan based on the changes in the anatomy in the beam's-eye-view projection. This proof-of-principle study demonstrated that autoplanning was possible and could emulate the quality of manual treatment planning. However, a deformed intensity map may not always be clinically deliverable. To reduce the likelihood for such a possibility, Ahunbay et al. [59] proposed a more sophisticated two-step procedure for auto-replanning. The first step uses an aperture morphing technique to transform MLC leaf segment based on anatomy changes. The second step applies a segment weight optimization, which reoptimizes the entire plan. The entire process takes 5–8 minutes, confirming the potential feasibility of such an approach. Continued improvement of auto-replanning, therefore, promises to make routine online adaptive radiotherapy a possibility. It may also reduce manual workload requirements for offline ART as well.

## 7. Conclusions

Feasible adaptive radiotherapy has long been a clinical goal. Current adaptive radiotherapy strategies remain labor and resource intensive. However, initial results from prospective clinical trial work employing automated deformable image registration demonstrate the feasibility and dosimetric benefit from use of head and neck adaptive radiotherapy. As ART clinical outcomes mature and incorporation of volumetric imaging into ART becomes increasingly sophisticated, we expect ART to evolve briskly towards becoming a commonplace approach for head and neck radiation treatment. Nonetheless, the optimal frequency and utilization as well as the ultimate clinical impact of ART remain undefined, and prospective clinical trials will be necessary to appropriately mold ART into a future treatment standard.
